# Urban amenity and settlement intentions of rural–urban migrants in China

**DOI:** 10.1371/journal.pone.0215868

**Published:** 2019-05-13

**Authors:** Liping Liao, Chunchao Wang

**Affiliations:** School of Economics, Jinan University, Guangzhou, China; Institute of Geographic Sciences and Natural Resources Research (IGSNRR), Chinese Academy of Sciences (CAS), CHINA

## Abstract

The existing literature concentrates on the relationship between amenities and migrants or residents. However, only a few studies have focused on the role of city amenities in determining the intentions of rural–urban migrants. Such a relation is a key issue in Chinese urbanisation development. The current study investigates the effects of urban amenities on the settlement intentions of rural–urban migrants in China. We find that medical amenities have a significantly positive effect on rural–urban migrants’ intentions. We also indicate that educational amenities and transportation services attract rural–urban migrants to settle in cities. Furthermore, we explore the heterogeneous effects of amenities on different cohorts by education and age. High- and low-skilled rural–urban migrants focus on transportation amenities, while young and middle–aged migrants are attracted by urban educational amenities. Results suggest that increasing access to urban amenities for rural–urban migrants and improving urban amenities enhance the willingness of rural–urban migrants to stay in cities.

## Introduction

In recent years, the number of rural–urban migrants has been steadily increasing and eventually reached 277.47 million in 2015 (a 1.3% increase from 2014). Although rural labourers often migrate to cities to make a living, they may not settle in the cities because of institutional and economic conditions. For example, *hukou* (household registration) impedes rural migrants from flowing freely to cities [1]. Farming income is negatively correlated with the probability of migration, while the least and most educated workers are likely to stay in their villages [2]. Human capital externalities discourage rural residents from moving to urban areas and exert a positive effect on their rural income [3]. Apart from the aforementioned determinants, the issue of whether the urban amenity environment affects residents’ willingness to stay in cities has rarely been investigated. Therefore, we explore how different amenities influence rural–urban migrants’ decision to settle in cities for a midterm period. We also determine whether these amenities have a heterogeneous influence on different cohorts.

Several studies have recognized the importance of the urban amenity environment to the well-being of residents, including rural–urban migrants [4–7]. Meanwhile, numerous studies have investigated the effects of urban amenities on rural migrants’ decisions [8–9]. The natural environment, including temperature and precipitation, and social and cultural amenities (e.g., availability of hospital beds and telephone lines) compensate for wages in the labour market [10–12]. The existing literature is concerned with the relationship between amenities and migration in China, but it has yet to consider midterm migration intentions [13]. Moreover, studies that explore the determinants of settlement intentions have paid little attention to regional environment characteristics. Although studies that use province-level data have considered amenities as influential factors for determining the location choices of migrants in China, these investigations do not focus on rural–urban migrants [9]. Our study extends the existing literature by mainly using city-level and individual data and focusing on the effects of urban amenities on the settlement intentions of rural–urban migrants in China.

We first establish a conceptual framework in which the utility of rural–urban migrants is determined by their consumption of traded composite commodities and non–traded housing services, thereby affecting their quality of life. Accordingly, rural–urban migrants may migrate to the destination when the indirect utility gaps between a destination and current residence are sufficiently large enough to cover migration costs. With a 0–1 indicator that measures rural migrants’ decisions to settle in a city, we use one-year lagged city-level data and estimate a probit model to obtain our basic results. Our empirical findings suggest that urban amenities significantly affect rural migrants’ decision to live in cities for a midterm period. That is, rural–urban migrants tend to live in cities with a reasonable number of hospitals and abundant teaching resources. Convenient transportation is also an important factor in their settlement decisions.

The remainder of this paper is organized as follows. Section 2 presents the literature review. Section 3 outlines our conceptual framework. Section 4 provides information on the data used. Section 5 describes our methodology. Section 6 presents the main results of this study and describes the robustness check. Section 7 concludes this research.

## Literature review

Several studies have addressed the determinants of the settlement of migrants in China. In examining samples from five large cities in China, the age of the oldest children in migrant families exerts considerably negative effects on their parents’ settlement patterns and that age and marriage factors show no correlation with migrant settlement patterns [14]. However, the relationship between age and the probability of permanent migration shows an inverse U-shaped pattern and highly educated and experienced migrants tend to become permanent urban residents [15]. Tang and Feng [16] compare the heterogeneities of two cohorts with different ages and find that young rural migrants have a strong desire to settle in urban areas. Self-employed migrants tend to harbour strong intentions to permanently settle in cities because they are closely integrated into city economies [13]. In addition, high housing prices are negatively correlated with settlement intentions, while access to formal housing plays a positive role in settlement intentions [17, 18].

In exploring the determinants of settlement patterns in China, the aforementioned studies are mainly focused on demographic characteristics, family-related factors, institutional factors, job features, housing access, and social security issues. Hence, the effects of regional environment characteristics on settlement decisions have been generally disregarded. Our research is expected to shed light on whether urban amenities are determinants of rural–urban migrants’ settlement intentions.

Despite the limited research on the impact of local amenities on Chinese migrants, scholars have noted the effects of urban amenities on quality of life measures and migration behaviour. Since the publication [11, 19], studies have explored the effects of city amenities on quality of life [8, 10, 12, 20]. Cao et al. [13] address natural amenities (e.g. temperature and cloudiness) and use hedonic prices to calculate the implicit prices of natural amenities, thereby regarding them as indicators of quality of life. Gyourko and Tracy [12] add fire control, education, police and medical spending measures to their model and find that these four aspects of financial expenditures can compensate for housing prices and wages. Albouy and Lue [21] create a willingness-to-pay index to reflect the quality of life that households receive from local amenities in the US, such as natural amenities and public education. By restricting a sample to urban residents, Marans [22] explores the measures of the quality of urban life, such as crime, school, traffic, and housing, from the objective and subjective aspects and suggests the importance of measures that create a culture of sustainability.

Local amenities affect the quality of life of individuals in cities and migrants’ intentions to migrate [8, 20, 23, 24]. Amenities have been identified as the main factors that influence migration patterns [5, 8, 9, 25, 26, 27]. Clark et al. [8] combine compensation for city amenities with migration behaviour and develop a three-stage model to test whether migration is influenced by incomplete wage compensation. These authors find that outmigration is unlikely to occur when a location is characterized by overcompensation. On the basis of a simple theoretical framework of regional utility, Rodríguez–Pose and Ketter [25] analyze the effects of various amenities (i.e., general city amenities, namely, cultural and artistic services, aesthetic goods and services, recreation and tourism and transportation and housing) on net population migration rates. For constraints on wealth, Dustmann and Okatenko [5] find that degrees of contentment with various local amenities (e.g., schools, education systems, health care systems, air quality, and security) substantially affect migration intentions in three geographic regions, namely, sub-Saharan Africa, Asia, and Latin America. Migrants are willing to settle in cities with many libraries, museums and medical doctors [9]. Neighbourhoods and lifestyles are special types of amenities that encourage individuals to migrate [28]. High school and college graduates in the US have different location choices, while the combination of desirable wages and amenities motivates college graduates’ preference for high-rent cities [29].

The existing literature related to city amenities, migration intentions and migration behaviour is concerned with other countries in Europe, Asia, Latin America, and Africa [8, 25]. Moreover, only a few studies have focused on the role of city amenities in determining the migration behaviour and intentions of rural–urban migrants in China. Excluding institutional factors [1], natural and social amenities also play an important role in the migration decisions of rural–urban migrants. Liu and Shen [9] use provincial-level data to examine the weights of amenities in determining skilled internal migrants’ destination choices in China. Our study is different from the aforementioned research, because we use city-level data to identify the effects of urban amenities on the midterm settlement intentions of migrants. This study focuses on rural–urban migrants by exploring the relationships between urban amenities and migrants’ settlement decisions. Thereafter, we use the heterogeneous results as bases to predict the type of urban amenity that is most beneficial in terms of attracting young and skilled migrants. Compared with current location choices, midterm settlement intentions can permanently reflect the job and life satisfaction of mobile rural migrants in cities. Hence, we focus on the roles of urban amenities in migrants’ settlement intentions.

## Conceptual framework

In the following models, as used by Roback [11], Rappaport [30] and Rodríguez–Pose and Ketter [25], a migrant’s utility is determined by traded composite consumption commodities, non–traded house services and a set of amenities. We present a settlement choice model that serves as the theoretical basis for our empirical analysis. Moreover, we use a utility maximization function for migrants to consider their intentions to settle in cities under budget constraints.

$$Max\;U_{ij}(G_{ij},H_{ij},S_{j},N_{j})$$ (1)

$$s.t.{\;G}_{ij}+P_{j}H_{ij}\;=\;I_{ij},$$

where *G*_*ij*_ and *H*_*ij*_ denote the quantity of traded composite commodities and non–traded house services, respectively, which migrant *i* will consume in city *j*. The price of *G*_*ij*_ is normalized to 1, while *P*_*j*_ denotes the housing price in city *j*. *S*_*j*_ refers to the urban social amenities of city *j*. When individual *i* chooses to stay in city *j*, such an individual would like to consume the social amenities provided in that city. *N*_*j*_ stands for the natural amenities, including climate, of city *j*. *I*_*ij*_ is the total income of individual *i* in city *j*.

Amenities determine the quality of life of individuals [12, 25]. Accordingly, we focus on various types of amenities (i.e., natural and social) and obtain the indirect utility function from the budget constraint and maximization Eq (1) as follows:

$$V_{ij}(P_{j},I_{ij},Z_{ij},S_{j},N_{j},\delta_{j}),$$ (2)

where ∂*V*_*ij*_/∂*P*_*j*_ < 0, ∂*V*_*ij*_/∂*I*_*ij*_ > 0; *δ*_*j*_ refers to the socioeconomic characteristics, such as per capita GDP, total population, and social climate; and *Z*_*ij*_ is a vector of the individual and household characteristics (e.g., age, age squared, marital status, gender, education level, occupation, industry, and family dependency ratio, which is calculated as the sum of the number of children under the age of 14 and the number of elderly people over the age of 65 divided by the family size). We are also interested in how the urban amenities of city *j* influence the utility of migrant *i*. We explore this topic in the later sections according to the empirical results.

As an indicator of well-being, net migration is used to measure the utility differentials between two cities [31]. From migrants’ point of view, a migration decision can reflect their life satisfaction in a city. If rural migrant *i*’s indirect utility differential between cities *j* and *k* is higher than the migration cost (if Δ*V*_*jk*_ − *C*_*jk*_ = *V*_*ik*_ − *V*_*ij*_ − *C*_*jk*_ > 0, *j* ≠ *k*), then migrant *i* will migrate to city *k* from city *j*; accordingly, *C*_*jk*_ denotes the migration costs when migrant *i* migrates from city *j* to city *k* and accounts for psychological, pecuniary, search and information costs. Pecuniary costs include forgone earnings and direct expenditures, while psychological costs emerge from the differences between living environments [32, 33]. Whether rural–urban migrants benefit from these amenities has yet to be determined. If the urban amenities of cities can improve the quality of life of rural–urban migrants, then they tend to settle in such cities. We discuss in detail the impact of amenities on rural–urban migrants in the following sections.

## Data and descriptive statistics

### Data

Our empirical analysis is based on the China Migrants Dynamic Survey (CMDS) data for 2012 provided by the Migrant Population Service Center of National Health Commission in China. Cross-sectional data involving employment, wage, and demographic information were collected from rural–urban migrants in cities. Given that this study focuses on the effects of urban amenities, we also use data drawn from the *China Urban Statistics Yearbook* on city amenities for 2011. Moreover, we obtain the commercial housing sales area and sales volume for each city from the *China Regional Economic Statistical Yearbook* for 2011. After matching these two datasets, we obtain 133073 samples that cover 230 prefecture-level cities and 31 provinces.

The CMDS dataset, which is randomly sampled on the basis of cities, provides a considerable amount of information on the floating population’s current conditions (e.g., data on household features, individual features, employment status, housing features, health care services, access to family planning services, lifestyles and happiness measures) and covers nearly all the provinces in China (including Xinjiang Autonomous Region). The survey respondents are members of a floating population aged 15 to 59 years, have lived in a city for at least one month or more, and hold a *hukou* of another county.

We use these data as follows. (1) “Cities” in this paper refer to municipal districts, but exclude counties and county-level cities, as municipal districts effectively reflect the features of the urban economy [34]. (2) Given that the consumer price index (CPI) of 2000 is assumed to be equal to 100, we convert income, per capita GDP, and housing prices to actual values on the basis of the provincial CPI because city-level CPI data are unavailable. (3) We focus on rural–urban migrants who obtain employment outside their villages and towns. In this paper, we define rural–urban migrants as those with rural *hukou* and have lived in cities for over one month.

### Descriptive statistics

#### Settlement intentions

The dependent variable used in this study is based on the responses to the following question: “Would you like to live in a city for at least five years?” The possible answers to this question are “Yes” and “No.” Overall, nearly 58.85% of rural–urban migrants are willing to live in a city for a midterm period. Detailed descriptions of the variables and data sources are presented in S1 Table. The descriptive statistics reported in Table 1 provide an overview of the samples.

**Table 1:** Descriptive statistics of the main variables.

Variables	Obs	Mean	Std.Dev.	Min	Max
**Panel A. Settlement intentions**					
Settlement intentions	133073	0.588	0.492	0	1
**Panel B. Control variables**					
Gender	133073	0.532	0.499	0	1
Marital status	133073	0.771	0.420	0	1
Dependency	133073	0.198	0.199	0	0.800
Work hours	111716	9.532	1.917	1	16
No schooling	133073	0.023	0.150	0	1
Elementary school	133073	0.159	0.366	0	1
Junior high school	133073	0.582	0.493	0	1
Senior high school	133073	0.142	0.349	0	1
Technical school	133073	0.051	0.220	0	1
Junior college and above	133073	0.034	0.181	0	1
Age	133073	33.440	9.337	15	60
State personnel	133073	0.002	0.041	0	1
Technical workers	133073	0.044	0.206	0	1
Public servants	133073	0.005	0.073	0	1
Business and service personnel	133073	0.481	0.500	0	1
Industrial workers	133073	0.245	0.430	0	1
Other	133073	0.030	0.170	0	1
Manufacturing	133073	0.180	0.384	0	1
Agriculture	133073	0.031	0.173	0	1
Extractive resources	133073	0.009	0.094	0	1
Building	133073	0.084	0.277	0	1
Industry of supply of water, coal and electricity	133073	0.005	0.067	0	1
Service	133073	0.531	0.499	0	1
Interprovincial movement	133073	0.434	0.496	0	1
Medical insurance	133073	0.154	0.360	0	1
ln total population	116629	5.507	1.081	2.728	7.479
ln per capita GDP	116380	4.312	0.481	2.393	5.901
ln real income	105895	7.539	0.559	4.233	11.180
ln real housing price	123196	-0.726	0.606	-2.135	0.526
The local people are willing to accept migrants as a member of them (acceptance)	132723	3.291	0.622	1	4
The local people look down upon migrants (equal treatment)	132552	2.988	0.791	1	4
ln industrial wastewater emissions	116387	3.720	0.996	0.295	7.346
ln industrial sulfur dioxide emissions (SO_2_)	115400	5.736	1.139	2.206	9.060
ln industrial smoke dust emissions	114734	4.825	1.350	1.414	10.32
**Panel C. Natural amenities**					
Annual average temperature in January	118881	1.120	8.206	-27.978	22.500
Annual average temperature in July	118881	26.710	3.486	12.500	32.600

#### Control variables

The individual-level characteristics that we used as controls are age, age squared, gender, educational attainment, occupation, industry, work hours, and logarithm of real income. We divided the educational attainment into the following six categories: no schooling, elementary school, junior high school, high school, technical school, and junior college and above. The following six occupations are considered: state personnel, technical personnel, public servant, business and service personnel, industrial worker, and others. The six industries are manufacturing; agriculture; extractive resources; construction; services; and supply of water, coal and electricity. Family size and the number of preschool children play an important role in determining whether rural–urban migrants intend to settle in a city. The family dependency ratio determines the potential fostering burdens within families.

Given the *hukou* restrictions on rural migrant workers, such individuals may not benefit from city medical services. Thus, we control for whether rural migrant workers have access to urban employee medical insurance. In controlling for the living and migration costs of rural migrant workers, we consider housing prices and whether a migrant’s places of origin and destination are in the same province [9]. We also control for the total population [8, 11] and per capita GDP.

Given that the quality of city environment is correlated with people’s migration choices, we select industrial sulfur dioxide emissions, industrial wastewater emissions, and industrial smoke dust emissions as measurements of environmental quality [11, 35]. The social climate of a city is one of the determinants of migrants’ location and migration decisions, such as openness and tolerance of the city. Consequently, we construct a social climate index with responses to the following two questions: “how much migrants feel that the local people are willing to accept them as a member of them” and “how much migrants feel that the local people look down upon them.” The answers are on a scale from 1 (*strongly disagree*) to 4 (*strongly agree*). Accordingly, we adjust the latter variable from a negative question to a positive one, namely, large values of a variable means a good social climate in the city. Thereafter, we use the principal component analysis method to group the two variables into one index.

Table 1 shows an equal ratio of males to females. Nearly half of the rural–urban migrants have a junior high school education, while their average age is 33.44 years. Nearly half of the migrant workers are engaged in businesses and services. The log of real income ranges from 4.23 to 11.18. The mean of the log of industrial wastewater emissions is 3.72, while industrial sulfur dioxide emissions range from 2.21 to 9.06.

#### Amenities

For the perspective of natural amenities, this study includes annual temperature in January and July as climate variables [8, 25]. Table 1 shows that the annual temperature in July varies from 12.5 °C to 32.6°C, while the mean of the annual temperature in January is 1.12 °C. For social amenity variables, we consider the three main factors of health care, education and transportation. Clark et al. [8] measure urban medical service quality based on medical expenditures, whereas Gyourko and Tracy [12] use the availability of hospital beds as a measure of urban public services. We combine the number of hospitals and the number of hospital beds per 10,000 people in evaluating a city’s medical services [35]. The descriptive statistics reported in Table 2 provide an overview of the samples used.

**Table 2:** Descriptive statistics of the social amenities.

Variables	Mean	S.D.
Number of hospitals per 10,000 people	0.517	0.496
Number of hospital beds per 10,000 people	72.442	19.666
Teacher-pupil ratio in elementary schools	0.056	0.011
Teacher-pupil ratio in junior and high schools	0.080	0.021
Number of buses per 10,000 people	13.932	16.286

Caselli [36] explain that the resources invested in education include teaching human capital, educational expenditures, and teacher–pupil ratio. However, Hanushek and Kimko [37] find that educational expenditures have no significant effect on student academic achievement. The educational level of teachers has no systematic relationship to teacher quality and student performance [38]. Therefore, we measure the quality of educational services for each city based on student–pupil ratios [12] by using two indicators: the ratio of junior to senior high school services and the ratio of elementary school services.

As an important component of urban social amenities, transportation affects migration behaviour. Berger et al. [10] show that commuting time compensates for wage differentials in cities and ultimately influences migration decisions. Poor populations tend to live in city centres to completely utilize convenient transportation services [39]. Given that the average wages of migrant workers are lower than those of urban workers, rural–urban migrants are likely to attach importance to public transportation services. We use the number of buses per 10,000 people to measure the quality of transportation services in a city.

## Empirical setup

Following Diamond [29], we measure amenity conditions using the first principal component of each social amenity category except in the transportation index. Table 3 reports the loadings of two social amenity indices, environment quality index and social climate index. All indices place positive loadings on their indicators; for example, the loading of the number of hospital beds per 10,000 people is 0.707, while the higher number of hospital beds reflects improved medical conditions. The education index positively weighs the teacher–pupil ratio for elementary school and junior and senior high school. The environmental pollution index positively weighs industrial wastewater emissions, industrial dust emissions and SO_2_. Accordingly, high emissions indicate severe pollution for each city. The social climate index positively weighs the acceptance and equal treatment of the local people.

**Table 3:** Principal component analysis of the amenity indices.

	Loading	Unexplained variance
**Panel A. Medical index**		
Number of hospital beds per 10,000 people	0.707	0.397
Number of hospitals per 10,000 people	0.707	0.397
**Panel B. Education index**		
Teacher-pupil ratio for junior and senior schools	0.707	0.297
Teacher-pupil ratio for elementary schools	0.707	0.297
**Panel C. Social climate index**		
The local people are willing to accept migrants as a member of them (acceptance)	0.707	0.341
The local people look down upon migrants (equal treatment)	0.707	0.341
**Panel D. Environmental pollution index**		
Industrial wastewater emissions	0.477	0.489
Industrial smoke dust emissions	0.610	0.164
Industrial SO_2_ emissions	0.633	0.098

Notes: The amenity data are measured in logs. See S1 Table for a detailed description of the amenity data and their sources.

Urban social amenities and settlement intentions may present issues of reciprocal causation, thereby resulting in endogeneity problems. Although urban social amenities are positively correlated with rural–urban migrants’ settlement decisions and location choices, it’s likely that the increasing rural–urban migrant workers improve the urban social amenities and promote a continuous urban economic development. Therefore, urban public service variables are lagged one year in the model to solve the potential endogenous problem [25, 40, 41]. Whether rural–urban migrants decide to settle in a city is determined on the basis of a 0–1 indicator. When rural–urban migrants are willing to settle in city *j*, the dependent variable *settle*_*ij*_ takes a value of 1; otherwise, its value is 0. To determine whether urban amenities affect the settlement decisions of rural–urban migrants, we estimate the probit specification as follows:

$$pr({settle}_{ij}\;=\;1)\;=\;pr(\Delta V_{jk}-C_{jk}>0)\;=\;\Phi (\alpha_{0}+\alpha_{H}lnP_{j}+\alpha_{H}lnI_{ij}+\beta_{Z}Z_{ij}+\beta_{C}{CL}_{j}+\beta_{M}M_{j}+\beta_{T}T_{j}+\beta_{E}E_{j}+\delta_{j}+\varepsilon_{ij}),$$ (3)

where *settle*_*ij*_ denotes the intention to settle (dummy variable); Φ is the cumulative probability distribution function of the standard normal; ln*P*_*j*_ is the log of real housing prices for city *j*; ln*I*_*ij*_ is the log of rural migrant *i*’s real income earned in city *j*; *M*_*j*_, *T*_*j*_, *E*_*j*_, *CL*_*j*_ represent medical resources, transportation services, educational resources, and climate conditions, respectively. *ε*_*ij*_ refers to the effect of unobserved variables. The parameter of interest in Eq (3) is *β*_*E*_, *β*_*M*_, *β*_*T*_, because it determines the effects of social amenities on rural–urban migrants’ intentions to settle. If the parameters are positive, then urban amenities are positively correlated with the settlement intentions of migrants.

## Results and robustness check

### Basic results

The results presented in Table 4 show that, as expected, all the variables for urban amenities are highly correlated with the settlement intentions of rural–urban migrant workers. Table 4 presents the estimates of the coefficient of interest, *β*_*E*_, *β*_*M*_, *β*_*T*_, *β*_*CL*_. Columns (1), (2), (3), and (4) show the probit estimates controlling for industry, occupation, and province fixed effect. All the urban amenity variables are included in the standard model in column (4). Education and transportation amenities have a significantly positive effect on settlement intentions (0.008 and 0.024, respectively). Meanwhile, rural–urban migrant workers are considerably willing to settle in places with a larger number of hospitals and hospital beds.

**Table 4:** The effects of urban amenities on rural-urban migrants’ settlement intentions calculated on the basis of probit regressions.

	(1)	(2)	(3)	(4)
Medical index	0.008***			0.006**
	(0.002)			(0.002)
Education index		0.007***		0.008***
		(0.002)		(0.003)
Transportation index			0.015***	0.024***
			(0.004)	(0.005)
Gender	0.002	0.000	0.001	0.001
	(0.003)	(0.003)	(0.003)	(0.003)
Marital status	0.062***	0.062***	0.061***	0.062***
	(0.006)	(0.006)	(0.005)	(0.006)
Dependency	0.140***	0.129***	0.139***	0.129***
	(0.011)	(0.010)	(0.010)	(0.011)
Work hours	0.003***	0.002***	0.003***	0.002***
	(0.001)	(0.001)	(0.001)	(0.001)
Elementary school	-0.002	-0.006	-0.003	-0.005
	(0.011)	(0.011)	(0.010)	(0.011)
Junior high school	-0.019*	-0.019*	-0.020**	-0.019*
	(0.010)	(0.010)	(0.010)	(0.010)
Senior high school	0.011	0.008	0.007	0.010
	(0.011)	(0.011)	(0.010)	(0.011)
Technical school	0.006	0.010	0.006	0.008
	(0.012)	(0.012)	(0.011)	(0.012)
Junior college and above	0.041***	0.039***	0.037***	0.041***
	(0.013)	(0.013)	(0.012)	(0.013)
Age	0.015***	0.016***	0.015***	0.016***
	(0.001)	(0.001)	(0.001)	(0.002)
Age squared	-0.000***	-0.000***	-0.000***	-0.000***
	(0.000)	(0.000)	(0.000)	(0.000)
Interprovincial movement	0.077***	0.069***	0.077***	0.067***
	(0.004)	(0.004)	(0.004)	(0.004)
Medical insurance	0.063***	0.062***	0.061***	0.061***
	(0.004)	(0.004)	(0.004)	(0.004)
ln total population	0.019***	0.018***	0.023***	0.017***
	(0.004)	(0.004)	(0.003)	(0.004)
ln per capita GDP	0.066***	0.082***	0.065***	0.074***
	(0.007)	(0.007)	(0.006)	(0.008)
ln real income	0.035***	0.038***	0.035***	0.039***
	(0.003)	(0.003)	(0.003)	(0.003)
ln real housing price	-0.004	-0.023**	-0.027***	-0.051***
	(0.009)	(0.009)	(0.010)	(0.011)
Annual average temperature in January	-0.000	0.000	-0.000	0.000
	(0.000)	(0.000)	(0.000)	(0.000)
Annual average temperature in July	-0.000**	-0.000	-0.000*	-0.000
	(0.000)	(0.000)	(0.000)	(0.000)
Social climate index	0.079***	0.079***	0.079***	0.079***
	(0.001)	(0.001)	(0.001)	(0.001)
Observations	85497	85497	85497	85497

Notes:

***p<0.01,

**p<0.05,

*p<0.1.

This table shows the marginal effects of probit regressions. The dependent variable is the settlement intentions of rural–urban migrants. Standard errors are indicated in parentheses. Industry, occupation, and province fixed effect are controlled in all the above regressions.

Cities with abundant educational resources are more attractive to rural–urban migrants. The settlement intentions of rural–urban migrants increase by 0.8% as the education index increases by one point. The higher number of teachers in urban schools can provide better learning environment for students. Thus, rural–urban migrants are more inclined to settle in cities in consideration of children’s human capital accumulation and future development. If the transportation increases by one point, the settlement intentions of rural–urban migrants rise significantly by 2.4%. According to our data, the average wage of migrant workers is 3,000 CNY, and nearly 72% of rural–urban migrants rent a house rather than live in their workplace. This fact suggests that the income of most rural–urban migrants is relatively low and they need to reduce housing costs; thus, they may live in the countryside or the city centre to benefit from public transportation by saving time and money. As Glaeser et al. [39] suggest, the poor tend to live in the city centre where transportation is convenient.

The coefficient of medical services ranges from 0.006 to 0.008; a city with a large number of doctors or hospital beds is the best choice for rural–urban migrants. After controlling whether rural–urban migrants have participated in medical insurance, we find that medical services have a positive effect on settlement intentions. Liu and Shen [9] draw a similar conclusion, finding that floating populations tend to settle in provinces with numerous doctors.

Not surprisingly, the log of income and housing prices is nearly significant for all regressions, and rural–urban migrants tend to settle in the cities with higher income and lower housing prices (0.039 and –0.051, respectively, as shown in column (4) of Table 4). Income and housing prices are important determinants of settlement decisions. The settlement intentions of married rural-urban migrants are 6.2% higher than unmarried migrants. The education level is correlated positively and significantly with the settlement intentions of rural–urban migrants. For example, rural–urban migrants with junior college and above degrees are more willing to settle down in cities (0.041).

City economic development is a critical determinant of rural–urban migrants’ settlement intentions. If the log of per capita GDP increases by 1 point, rural–urban migrants’ settlement intentions rise by 7.4%. Large cities are more attractive for rural–urban migrants, and the coefficient of the log of population is 0.017, which is significant at the 1% level. The social climate index displaces a positive correlation with rural-urban migrants’ settlement intentions, which means these migrants prefer living in cities with higher openness and tolerance. Considering that the city environment quality and pollution level may affect rural–urban migrants’ settlement decisions [25, 35], we add an environmental quality index and estimate the Eq (3) again. The results are shown in S2 Table. The results show that when environmental pollution level of cities is controlled, the coefficients are almost the same.

### Heterogeneous effect

In this section, we aim to identify the effect of urban social amenities on different cohorts and analyze the heterogeneous effect of amenities by rural–urban migrants’ skill levels and age. In the US, high-skilled workers tend to concentrate in high–productivity cities, which leads to a further increase in local productivity and workers’ wages as well as an improvement in city amenities [29]. We investigate whether the skill–sorting phenomenon exists among rural–urban migrants in China and whether high–skilled rural–urban migrants tend to live in cities with higher wages and improved amenities. Although Diamond [29] classifies workers with four–year university degrees as high-skilled, we define high-skilled workers as those with senior high school and above degrees because rural–urban migrants usually receive less education than urban local workers in China.

The regression results of low–skilled and high–skilled rural–urban migrants are shown in columns (1) and (2) of Table 5. Both high–skilled and low–skilled migrants tend to settle in cities with higher wages, and high–skilled rural migrants are more sensitive to income. If the log of income adds one point, then the settlement intentions of rural–urban migrants increase by 3.3% for low–skilled workers and 5.6% for high–skilled workers. Low–skilled workers are willing to stay in cities with lower housing prices, and housing prices have a positive but insignificant effect on high–skilled rural–urban migrants.

**Table 5:** Heterogeneous effect of urban amenities on rural–urban migrants by age and skill.

	(1)	(2)	(3)	(4)	(5)
	Low-skilled	High-skilled	Aged 16–24	Aged 25–45	Aged 46–59
Medical index	0.005*	0.015***	0.012*	0.004	0.000
	(0.003)	(0.006)	(0.007)	(0.003)	(0.007)
Education index	0.009***	0.007	0.015**	0.006**	0.008
	(0.003)	(0.006)	(0.007)	(0.003)	(0.007)
Transportation index	0.021***	0.031***	0.009	0.024***	0.017
	(0.006)	(0.012)	(0.013)	(0.006)	(0.018)
ln real income	0.033***	0.056***	0.051***	0.045***	0.004
	(0.004)	(0.007)	(0.010)	(0.004)	(0.009)
ln real housing price	-0.056***	0.015	-0.024	-0.057***	-0.029
	(0.013)	(0.030)	(0.028)	(0.013)	(0.033)
Gender	0.001	-0.007	0.008	-0.004	0.010
	(0.004)	(0.007)	(0.008)	(0.004)	(0.011)
Marital status	0.051***	0.076***	0.059***	0.059***	0.063***
	(0.007)	(0.011)	(0.016)	(0.007)	(0.020)
Dependency	0.129***	0.121***	0.172***	0.122***	0.250***
	(0.012)	(0.025)	(0.054)	(0.012)	(0.045)
Work hours	0.003***	-0.001	-0.001	0.003***	0.005**
	(0.001)	(0.002)	(0.002)	(0.001)	(0.002)
Elementary school			0.016	-0.006	0.000
			(0.039)	(0.013)	(0.024)
Junior high school			0.018	-0.029**	0.013
			(0.032)	(0.012)	(0.024)
Senior high school			0.050	-0.005	0.077***
			(0.032)	(0.013)	(0.027)
Technical school			0.027	0.013	0.085
			(0.033)	(0.015)	(0.070)
Junior college and above			0.045	0.045***	0.019
			(0.034)	(0.016)	(0.083)
Age	0.017***	0.013***	-0.072**	0.020***	-0.006
	(0.002)	(0.003)	(0.035)	(0.004)	(0.036)
Age squared	-0.000***	-0.000**	0.002**	-0.000***	0.000
	(0.000)	(0.000)	(0.001)	(0.000)	(0.000)
Interprovincial movement	0.068***	0.064***	0.068***	0.066***	0.070***
	(0.005)	(0.008)	(0.010)	(0.005)	(0.012)
Medical insurance	0.061***	0.055***	0.035***	0.070***	0.043***
	(0.006)	(0.008)	(0.010)	(0.005)	(0.015)
ln total population	0.018***	0.017*	0.029***	0.017***	0.003
	(0.005)	(0.010)	(0.011)	(0.005)	(0.013)
ln per capita GDP	0.076***	0.042**	0.100***	0.071***	0.057***
	(0.009)	(0.017)	(0.020)	(0.009)	(0.022)
Annual average temperature in January	0.000	0.000*	0.000	0.000	-0.001*
	(0.000)	(0.000)	(0.000)	(0.000)	(0.000)
Annual average temperature in July	-0.000	-0.001*	-0.000	-0.000	-0.000
	(0.000)	(0.000)	(0.000)	(0.000)	(0.001)
Social climate index	0.080***	0.076***	0.076***	0.080***	0.075***
	(0.002)	(0.003)	(0.003)	(0.002)	(0.004)
Observations	63607	21890	15553	60394	9550

Notes:

***p<0.01,

**p<0.05,

*p<0.1.

The table shows the marginal effect of probit regressions. The dependent variable is the settlement intentions of rural-urban migrants. Standard errors are indicated in parentheses. Industry, occupation, and province fixed effect are controlled in all the above regressions.

The settlement intentions of the two groups rise as the medical index increases. High–skilled rural–urban migrants are more sensitive to medical resources, paying closer attention to health status. A city with numerous teachers significantly attract low–skilled rural–urban migrants, but high–skilled migrants are not sensitive to this amenity. The possible reasons are first, low–skilled migrants pay closer attention to the accumulation of human capital by learning, and second, low–skilled rural–urban migrants are inclined to settle in a city with abundant educational resources to ensure the future development of their children. The settlement intentions of low–skilled rural–urban migrants increase by 2.1% when the transportation index increases by one point, while the settlement intentions of high-skilled rural–urban migrants increase by 3.1%. For low–skilled workers, social climate plays a more important role in their settlement intentions. Their settlement intentions rise by 0.080 if the social climate index increases by 1 point. Low–skilled workers are more willing to settle in cities with a higher level of economic development, and the effect size of city per capita GDP is 0.076.

We categorize rural–urban migrants into three groups according to their age, defining young workers as those aged 16–24 years, old workers as those aged 46–59 years and middle–aged workers as those aged 25–45 years. The results are listed in columns (3), (4) and (5) of Table 5. The results show both young and middle–aged groups are significantly inclined to stay in cities with a higher income, and only the middle–aged group is influenced significantly and negatively by housing prices. Although both young and middle–aged migrants responded positively to the educational amenities in a city, the coefficients of the young migrants are relatively higher. Young workers place greater emphasis on medical resources, and middle–aged migrant workers tend to live in comfortable and convenient cities. The coefficient of transportation index for the middle–aged group is 0.024, which is significant at the 1% level. Old–aged migrant workers are not sensitive to urban amenities. Although urban social amenities are correlated positively with old migrant workers’ settlement intentions, it is not statistically significant.

The economic development of cities is an important determinant for young and middle–aged workers to make a settlement decision. If the log of per capita GDP rises by 1 point, the settlement intentions will increase by 10% for young workers and 7.1% for the middle–aged group. The social climate index shows a positive correlation with rural–urban migrant workers’ settlement intentions at each age group. Rural–urban migrant workers prefer living in cities with higher openness, which can help them integrate into cities and improve their satisfaction.

### Robustness check

**Fig 1:**
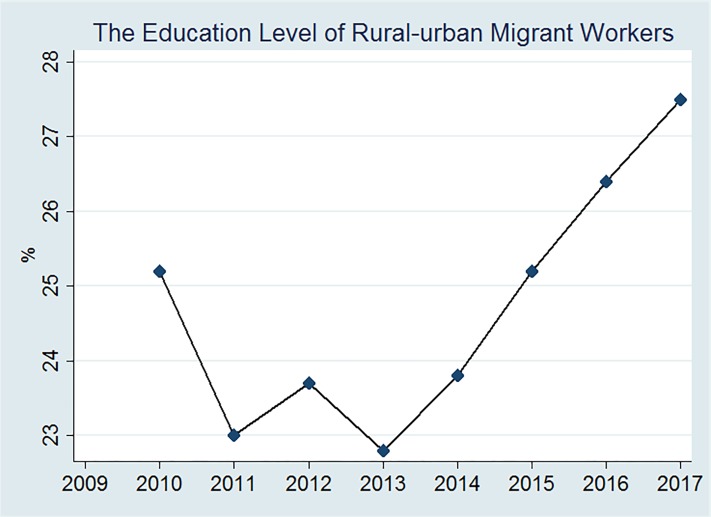
Rural-urban migrants’ education level. Data source: National Bureau of Statistics.

The paper focuses on rural–urban migrants’ settlement intentions, it may be a puzzle why rural–urban migrants place emphasis on urban educational amenities, because their children may be less likely to attend the local public schools for *hukou* restrictions. To explore this issue further, we first collected data from National Bureau of Statistics, and found that the education level of rural–urban migrants are increasing from 2009 to 2017, and the number of rural–urban migrants with senior high school or above degrees are nearly 24% in 2012 (see Fig 1). As rural–urban migrant workers’ education level increases, they will give more attention to the educational amenities of cities.

**Fig 2:**
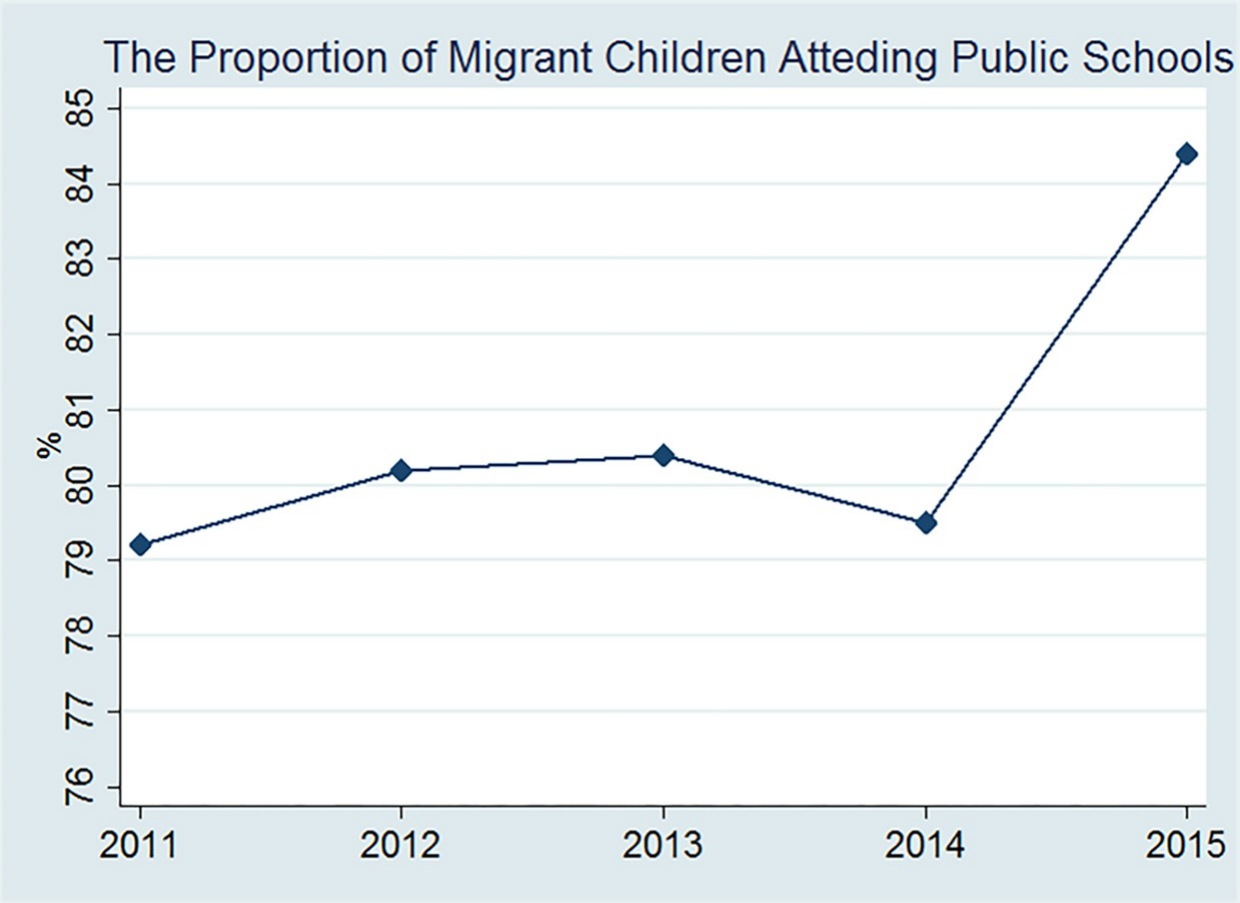
The proportion of rural–urban migrants’ children attending public schools. Data source: Ministry of Education in China.

Second, since the new Compulsory Education Law was implemented in 2006, the local government has provided equal access to compulsory education for the rural–urban migrants’ children. At present, the enrolment problem of migrant children is solved with the “two-oriented” policy, which means that rural-urban migrants’ children usually attend the local urban schools and most of them can attend the public schools. Based on data from Ministry of Education in China, we find that an increasing number of rural-urban migrants’ children attend the local public schools, and the proportion is more than 80% in 2012 (see Fig 2). Fig 2 shows that, most rural–urban migrants’ children attend the local urban public schools, and so migrants will take the educational amenities of cities into account when they make a settlement decision.

Third, accounting for the differences in economic development and migration restrictions among provinces, the proportion of migrant children who attend local public schools should be at least more than 50%. In Beijing, for the example, the proportion of migrant children attending public schools in 2011 was 70%. In Shanghai, a city with a high level of economic development, the proportion reached 74.72% in 2012, according to the data from Ministry of Education in China. Therefore, urban social amenities, including medical, educational and transportation amenities play an important role in rural–urban migrants’ settlement decisions. In China, the level of difficulty for rural–urban migrants’ children to attend public schools in cities is usually correlated with economic development and the size of cities. Considering the differences of enrolment restrictions for migrant children among cities, we run the regression excluding several large cities (Guangzhou, Shenzhen, Beijing and Shanghai) to obtain more robust results. The results in S3 Table show that urban social amenities are still associated positively with rural–urban migrants’ settlement intentions when the sample in large cities is excluded, and the coefficients change slightly. For example, the coefficient of education index rises from 0.008 to 0.011.

To assess whether the coefficients of the model are sensitive to estimation methods and regression models, we re-estimate the model using the OLS approach. These results are shown in S4 Table. In sum, the results are similar even when the estimation method or regression model changes. In addition, to test whether PCA is used reasonably, we create a social amenity index that includes all the social amenity variables. Then, we add all social amenity variables separately in the model. The results listed in S4 Table report that the urban social amenity index is correlated significantly with the settlement intentions of rural–urban migrants. Further details are provided in S4 Table.

## Conclusions

Using CMDS data for 2012, we examine whether urban social amenities affect the decisions of Chinese rural-urban migrant workers to live in cities for relatively long periods (at least five years). We conclude that social amenities are correlated significantly with rural–urban migrants’ intentions to settle in cities. These migrants are more inclined to live in cities with abundant educational resources, medical resources and convenient transportation.

In addition, we analyze the heterogeneous effect of urban amenity environment on rural–urban migrants categorized by age and skill levels. Like that in the US, high–skilled rural migrants in China usually concentrate in cities with higher incomes and rents, although the difference is statistically insignificant. By contrast, low–skilled rural migrants are more willing to stay in high–income, low–rent cities and low–skilled rural migrants place more weight on better educational resources. Transportation shows a positive effect on all rural–urban migrants, but is significant only for the middle–aged group. Both young and middle–aged migrant workers regard educational amenity as an important determinant of their settlement intentions. To motivate rural–urban migrants to stay steadily in cities, the government should help rural–urban migrants get more access to urban social amenities, improve the equalization of urban public services, and reduce or eliminate the restrictions for rural–urban migrant workers to use urban public services. Improving the urban amenity environment and increasing investments in education, medical treatment and transportation can also strengthen the attractiveness of cities. Creating an open and tolerant social climate of cities will also make a big difference in attracting rural–urban migrants.

## Supporting information

S1 Table Data description and data sources. (DOCX)

S2 Table The effect of urban social amenities on settlement intentions of rural-urban migrants after controlling the city environment pollution level. (DOCX)

S3 Table The effect of urban social amenities on rural-urban migrants’ settlement intentions excluding the sample of large cities. (DOCX)

S4 Table Additional robustness tests. (DOCX)
