# Supplementary material for: Urban amenity and settlement intentions of rural–urban migrants in China
Source: PLoS One. 2019 May 13;14(5):e0215868. doi: 10.1371/journal.pone.0215868 (PMC6513265; doi:10.1371/journal.pone.0215868)
Supplement: S1 Table — (DOCX) [file pone.0215868.s001.docx]

**S1 Table. Data description and data sources**

| Variable | Description | Source |
| --- | --- | --- |
| Settlement intentions | Dummy variable. Settlement=1 if rural migrant workers are willing to settle in residential cities | China Migrants Dynamic Survey data for 2012 |
|  |  |  |
|  |  |  |
| Work hours | Work hours for one day. | China Migrants Dynamic Survey data for 2012 |
| Gender | Male=1, female=0 | China Migrants Dynamic Survey data for 2012 |
| Education | Six education levels are considered: no schooling, elementary school, junior high school, high school, technical school, junior college and above. We add these to the model as dummy variables. | China Migrants Dynamic Survey data for 2012 |
| Age | The age of individuals. | China Migrants Dynamic Survey data for 2012 |
| Marital status | Dummy variable. If the individual is married, marital status=1. | China Migrants Dynamic Survey data for 2012 |
| Industry | Six industries are considered: manufacturing; agriculture; extractive resources; building; services; and the industry of supply of water, coal and electricity. We add these to the model as dummy variables. | China Migrants Dynamic Survey data for 2012 |
|  |  |  |
|  |  |  |
| Occupation | Six occupations are considered: state personnel, technical positions, public servants, business and services personnel, industrial workers and others. We add these to the model as dummy variables. | China Migrants Dynamic Survey data for 2012 |
|  |  |  |
|  |  |  |
| Dependency | The ratio equals the sum of children below the age of 14 and elderly over the age of 65 divided by family size. | Calculation |
| Medical insurance | Dummy variable. If the individual benefits from urban medical insurance, medical insurance=1. | China Migrants Dynamic Survey data for 2012 |
| Ln (income) | Log of real income. | China Migrants Dynamic Survey data for 2012 |
| The local people are willing to accept migrants as a member of them | 1=strongly disagree; 2= disagree; 3=agree; 4= strongly agree | China Migrants Dynamic Survey data for 2012 |
| The local people look down upon migrants | 1=strongly disagree; 2= disagree; 3=agree; 4= strongly agree | China Migrants Dynamic Survey data for 2012 |
| Ln (housing price) | Log of real housing price. | China Regional Economic Statistical Yearbook |
| Total population | The total population in the municipal district. | China Urban Statistics Yearbook |
| Per capita GDP | Annual GDP divided by the total population of the municipal district. | China Urban Statistics Yearbook |
| Number of hospitals per 10,000 people in 2011 | The number of hospitals per 10,000 people in the municipal district. | China Urban Statistics Yearbook |
| Number of hospital beds per 10,000 people in 2011 | The number of hospital beds per 10,000 people in the municipal district. | China Urban Statistics Yearbook |
| Teacher-pupil ratio for elementary schools in 2011 | The teacher-pupil ratio in elementary schools in the municipal district. | China Urban Statistics Yearbook |
| Teacher-pupil ratio for junior high and high schools in 2011 | The teacher-pupil ratio in junior high and high schools in the municipal district. | China Urban Statistics Yearbook |
| Number of buses per 10,000 people in 2011 | The number of buses per 10,000 people in the municipal district. | China Urban Statistics Yearbook |
| Temperature in January | The annual average temperature in January in degrees Celsius. | China Meteorological Bureau |
| Temperature in July | The annual average temperature in July in degrees Celsius. | China Meteorological Bureau |
| Industrial wastewater emissions | Industrial wastewater emissions per capita in ten thousand tons in the municipal district. | China Urban Statistics Yearbook |
| Industrial smoke dust emissions | Industrial smoke dust emissions per capita in tons in the municipal district. | China Urban Statistics Yearbook |
| Industrial SO_2_ emissions | Industrial sulfur dioxide emissions per capita in tons in the municipal district. | China Urban Statistics Yearbook |
