# Supplementary material for: Urban amenity and settlement intentions of rural–urban migrants in China
Source: PLoS One. 2019 May 13;14(5):e0215868. doi: 10.1371/journal.pone.0215868 (PMC6513265; doi:10.1371/journal.pone.0215868)
Supplement: S2 Table — (DOCX) [file pone.0215868.s002.docx]

**S2 Table. The effect of urban social amenities on settlement intentions of rural-urban migrants after controlling the city environment pollution level**

|  | (1) | (2) | (3) | (4) |
| --- | --- | --- | --- | --- |
| Medical index | 0.013^***^ |  |  | 0.010^***^ |
|  | (0.002) |  |  | (0.003) |
| Education index |  | 0.008^***^ |  | 0.010^***^ |
|  |  | (0.002) |  | (0.003) |
| Transportation index |  |  | 0.015^***^ | 0.028^***^ |
|  |  |  | (0.004) | (0.006) |
| Gender | 0.002 | 0.000 | 0.002 | 0.001 |
|  | (0.003) | (0.003) | (0.003) | (0.003) |
| Marital status | 0.062^***^ | 0.063^***^ | 0.061^***^ | 0.062^***^ |
|  | (0.006) | (0.006) | (0.005) | (0.006) |
| Dependency | 0.137^***^ | 0.127^***^ | 0.135^***^ | 0.128^***^ |
|  | (0.011) | (0.011) | (0.010) | (0.011) |
| Work hours | 0.003^***^ | 0.002^***^ | 0.003^***^ | 0.002^***^ |
|  | (0.001) | (0.001) | (0.001) | (0.001) |
| Elementary school | -0.002 | -0.006 | -0.003 | -0.005 |
|  | (0.011) | (0.011) | (0.010) | (0.011) |
| Junior high school | -0.020^**^ | -0.020^**^ | -0.021^**^ | -0.021^**^ |
|  | (0.010) | (0.010) | (0.010) | (0.010) |
| Senior high school | 0.012 | 0.008 | 0.007 | 0.010 |
|  | (0.011) | (0.011) | (0.010) | (0.011) |
| Technical school | 0.007 | 0.010 | 0.006 | 0.008 |
|  | (0.012) | (0.012) | (0.011) | (0.012) |
| Junior college and above | 0.040^***^ | 0.038^***^ | 0.036^***^ | 0.040^***^ |
|  | (0.013) | (0.013) | (0.012) | (0.013) |
| Age | 0.016^***^ | 0.016^***^ | 0.016^***^ | 0.016^***^ |
|  | (0.001) | (0.001) | (0.001) | (0.002) |
| Age squared | -0.000^***^ | -0.000^***^ | -0.000^***^ | -0.000^***^ |
|  | (0.000) | (0.000) | (0.000) | (0.000) |
| Interprovincial movement | 0.078^***^ | 0.069^***^ | 0.077^***^ | 0.068^***^ |
|  | (0.004) | (0.004) | (0.004) | (0.004) |
| Medical insurance | 0.063^***^ | 0.062^***^ | 0.062^***^ | 0.061^***^ |
|  | (0.004) | (0.004) | (0.004) | (0.004) |
| ln total population | 0.027^***^ | 0.021^***^ | 0.028^***^ | 0.024^***^ |
|  | (0.004) | (0.004) | (0.004) | (0.005) |
| ln per capita GDP | 0.047^***^ | 0.068^***^ | 0.052^***^ | 0.053^***^ |
|  | (0.007) | (0.007) | (0.006) | (0.008) |
| ln real income | 0.036^***^ | 0.039^***^ | 0.037^***^ | 0.039^***^ |
|  | (0.003) | (0.003) | (0.003) | (0.003) |
| ln real housing price | 0.014 | -0.007 | -0.022^*^ | -0.033^**^ |
|  | (0.010) | (0.011) | (0.011) | (0.014) |
| Annual average temperature in January | 0.000 | 0.000 | -0.000 | 0.000 |
|  | (0.000) | (0.000) | (0.000) | (0.000) |
| Annual average temperature in July | -0.000 | 0.000 | -0.000 | -0.000 |
|  | (0.000) | (0.000) | (0.000) | (0.000) |
| Social climate index | 0.079^***^ | 0.079^***^ | 0.079^***^ | 0.079^***^ |
|  | (0.001) | (0.001) | (0.001) | (0.001) |
| Environment pollution index | 0.011^***^ | 0.008^***^ | 0.006^***^ | 0.012^***^ |
|  | (0.003) | (0.003) | (0.002) | (0.003) |
| Observations | 84201 | 84201 | 84201 | 84201 |

Notes: ^***^p<0.01, ^**^p<0.05, ^*^p<0.1. The dependent variable is the settlement intentions of rural-urban migrants. Standard errors are indicated in parentheses. Industry, occupation, and province fixed effect are controlled in all the above regressions.
