# Supplementary material for: Urban amenity and settlement intentions of rural–urban migrants in China
Source: PLoS One. 2019 May 13;14(5):e0215868. doi: 10.1371/journal.pone.0215868 (PMC6513265; doi:10.1371/journal.pone.0215868)
Supplement: S3 Table — (DOCX) [file pone.0215868.s003.docx]

**S3 Table. The effect of urban social amenities on rural-urban migrants’ settlement intentions excluding the sample of large cities**

|  | (1) | (2) |
| --- | --- | --- |
| Medical index | 0.009^***^ | 0.012^***^ |
|  | (0.003) | (0.003) |
| Education index | 0.009^***^ | 0.011^***^ |
|  | (0.003) | (0.003) |
| Transportation index | 0.046^***^ | 0.049^***^ |
|  | (0.007) | (0.007) |
| Gender | -0.002 | -0.002 |
|  | (0.004) | (0.004) |
| Marital status | 0.072^***^ | 0.072^***^ |
|  | (0.007) | (0.007) |
| Dependency | 0.129^***^ | 0.127^***^ |
|  | (0.013) | (0.013) |
| Work hours | 0.004^***^ | 0.004^***^ |
|  | (0.001) | (0.001) |
| Elementary school | -0.005 | -0.005 |
|  | (0.013) | (0.013) |
| Junior high school | -0.026^**^ | -0.028^**^ |
|  | (0.012) | (0.012) |
| Senior high school | 0.001 | 0.002 |
|  | (0.013) | (0.013) |
| Technical school | 0.007 | 0.008 |
|  | (0.014) | (0.015) |
| Junior college and above | 0.035^**^ | 0.033^**^ |
|  | (0.016) | (0.016) |
| Age | 0.015^***^ | 0.015^***^ |
|  | (0.002) | (0.002) |
| Age squared | -0.000^***^ | -0.000^***^ |
|  | (0.000) | (0.000) |
| Interprovincial movement | 0.068^***^ | 0.069^***^ |
|  | (0.004) | (0.004) |
| Medical insurance | 0.071^***^ | 0.071^***^ |
|  | (0.005) | (0.006) |
| ln total population | -0.001 | 0.004 |
|  | (0.005) | (0.006) |
| ln per capita GDP | 0.057^***^ | 0.042^***^ |
|  | (0.008) | (0.009) |
| ln real income | 0.028^***^ | 0.028^***^ |
|  | (0.004) | (0.004) |
| ln real housing price | -0.027^**^ | -0.021 |
|  | (0.012) | (0.014) |
| Annual average temperature in January | 0.000 | 0.000 |
|  | (0.000) | (0.000) |
| Annual average temperature in July | 0.000 | 0.000 |
|  | (0.000) | (0.000) |
| Social climate index | 0.087^***^ | 0.087^***^ |
|  | (0.002) | (0.002) |
| Environment pollution index |  | 0.006^*^ |
|  |  | (0.003) |
| Observations | 66744 | 65448 |

Notes: ^***^p<0.01, ^**^p<0.05, ^*^p<0.1. The dependent variable is the settlement intentions of rural-urban migrants. Standard errors are indicated in parentheses. In column (1), we add the same list of control variables as in Table 4. In column (2), the environmental quality of cities is also controlled. Industry, occupation, and province fixed effect are controlled in all the above regressions.
