# Supplementary material for: Urban amenity and settlement intentions of rural–urban migrants in China
Source: PLoS One. 2019 May 13;14(5):e0215868. doi: 10.1371/journal.pone.0215868 (PMC6513265; doi:10.1371/journal.pone.0215868)
Supplement: S4 Table — (DOCX) [file pone.0215868.s004.docx]

**S4 Table. Additional robustness tests**

|  | (1) | (2) | (3) |
| --- | --- | --- | --- |
| Social climate index | 0.078^***^ | 0.081^***^ | 0.078^***^ |
|  | (0.001) | (0.001) | (0.001) |
| Medical index | 0.011^***^ |  |  |
|  | (0.002) |  |  |
| Education index | 0.007^***^ |  |  |
|  | (0.002) |  |  |
| Transportation index | 0.031^***^ |  | 0.011^***^ |
|  | (0.004) |  | (0.004) |
| Social amenity index |  | 0.011^***^ |  |
|  |  | (0.002) |  |
| Number of hospital beds per 10,000 people |  |  | 0.003 |
|  |  |  | (0.007) |
| Number of hospitals per 10,000 people |  |  | 0.025^***^ |
|  |  |  | (0.004) |
| Teacher-pupil ratio for junior high and high schools |  |  | 0.043^***^ |
|  |  |  | (0.009) |
| Teacher-pupil ratio for elementary schools |  |  | 0.000 |
|  |  |  | (0.012) |
| Observations | 85497 | 85497 | 85497 |
| adj. *R*^2^ | 0.109 |  |  |

Notes: ^***^p<0.01, ^**^p<0.05, ^*^p<0.1. The dependent variable is the settlement intentions of rural-urban migrants. Standard errors are indicated in parentheses. We add the same list of control variables as in Table 4. In column (1), we run the regression with OLS. In column (2), all the social amenity variables are integrated into an overall social amenity index. In column (3), all the social amenity variables are added to the equation separately. Industry, occupation, and province fixed effect are controlled in all the above regressions.
